# Intergeneric transfer of ribosomal genes between two fungi

**DOI:** 10.1186/1471-2148-8-87

**Published:** 2008-03-18

**Authors:** Jiatao Xie, Yanping Fu, Daohong Jiang, Guoqing Li, Junbin Huang, Bo Li, Tom Hsiang, Youliang Peng

**Affiliations:** 1 The Key Lab of Plant Pathology of Hubei Province, College of Plant Science and Technology, Huazhong Agricultural University, Wuhan 430070, Hubei, P R China; 2 National Key Lab of Agricultural Microbiology, Huazhong Agricultural University, Wuhan 430070, Hubei, P R China; 3 Department of Environmental Biology, University of Guelph, Guelph, ON, Canada; 4 Department of Plant Pathology, China Agricultural University, Beijing, 100094, P R China

## Abstract

**Background:**

Horizontal gene transfer, also called lateral gene transfer, frequently occurs among prokaryotic organisms, and is considered an important force in their evolution. However, there are relatively few reports of transfer to or from fungi, with some notable exceptions in the acquisition of prokaryotic genes. Some fungal species have been found to contain sequences resembling those of bacterial genes, and with such sequences absent in other fungal species, this has been interpreted as horizontal gene transfer. Similarly, a few fungi have been found to contain genes absent in close relatives but present in more distantly related taxa, and horizontal gene transfer has been invoked as a parsimonious explanation. There is a paucity of direct experimental evidence demonstrating the occurrence of horizontal gene transfer in fungi.

**Results:**

We found a fungal field isolate from rice (*Oryzae sativa*) that contains ribosomal DNA sequences from two species of fungal rice pathogens (*Thanatephorus cucumeris *and *Ceratobasidium oryzae-sativae*). This field isolate has four types of ribosomal DNA internal transcribed spacers (ITS), namely pure ITS of *C. oryzae-sativae*, which was dominant in this field isolate, pure ITS of *T. cucumeris*, and two chimeric ITS, with ITS1 derived from *C. oryzae-sativae *and ITS2 from *T. cucumeris*, or ITS1 from *T. cucumeri*s and ITS2 from *C. oryzae-sativae*. The presence of chimeric forms indicates that the intergeneric hybrid was not merely composed of nuclei from the parental species, but that nuclear fusion and crossing over had taken place.

**Conclusion:**

Hyphae of *T. cucumeris *and *C. oryzae-sativae *are vegetatively incompatible, and do not successfully anastomose. However, they parasitize the same host, and perhaps under the influence of host enzymes targeted to weaken pathogen cells or in dying host plant tissue, the fungal hyphae lost their integrity, and normal vegetative incompatibility mechanisms were overcome, allowing the hyphae to fuse. Based on the presence of other similarly anomalous isolates from the field, we speculate that these types of intergeneric hybridization events and occurrences of horizontal gene transfer may not be so rare in the field.

## Background

Horizontal gene transfer (HGT), also called lateral gene transfer, is known to occur among prokaryotic organisms, and is considered an important force in their evolution [1], but HGT is less well known among eukaryotes [2]. Based on a study of known protein domain families and publicly available sequences, there is evidence for HGT among more than 50% of archaea, between 30% to 50% of bacteria, and less than 10% of eukaryotes [3].

The role of HGT in the evolution of fungi was recently reviewed [4], and the authors speculated that the mode of feeding and large surface area in association with other organisms allow fungi a greater opportunity for HGT than other eukaryotes. Among fungi, some species have been found to contain sequences resembling those of bacterial genes, but such sequences were absent in other fungal species, and this was interpreted as HGT [5-10]. Transfer of a supernumerary chromosome between vegetatively incompatible biotypes of the phytopathogenic fungus, *Colletotrichum gloeosporioides*, was obtained under laboratory conditions [11]. Gene clusters encoding for epipolythiodioxopiperazines, which are a class of secondary metabolite toxins, have been found in various ascomycete fungi, but the phylogeny of these gene clusters was not concordant with that of the organisms in which they were found. [12]. A nitrate assimilation gene cluster was thought to have been transferred first from Oomycota to Dikarya (Ascomycota and Basidiomycota), and then more recently from a basdiomycete to the ascomycete *Trichoderma reesei*, which allowed this fungus to better exploit its new niche [13].

In a review of the evolution of virulence genes in fungi [14], the authors speculate that HGT is important in their origin in different pathogenic species, and may facilitate the emergence of new pathogens. They also noted the phenomenon where virulence genes are present in clusters. For example, a cluster of genes similar to those in pea was found on a supernumerary chromosome of three different fungi pathogenic to pea [15]. The authors of the review also speculate that the high density of transposable and repetitive elements frequently observed near putative virulence genes somehow allows or even facilitates the clustering of virulence genes, and that regions characterised by particular sequences may be more susceptible to HGT.

One group of genes that has not been demonstrated to be subjected to HGT encodes for ribosomal RNA [3]. These genes are found in the ribosomal DNA (rDNA) region, and form what has been called the ribosomal cassette. The rRNA combine with protein molecules to form ribosomes that function in protein synthesis. Because of the high demand for protein synthesis, there are multiple copies repeated tens to thousands of times in a genome [16]. Eukaryotes have rDNA cassettes formed by the small subunit (18S), the internally transcribed spacer (ITS) 1, the 5.8S gene, the ITS2, the large subunit (28S) and the intergenic spacer region. The differences between rDNA sequences allow for studies of phylogenetic relationships over a wide range of taxonomic levels [17].

In the current study, we found that DNA of a field fungal strain from rice (*Oryzae sativa*), was amplified with two sets of specific, normally diagnostic rDNA primers: one set for the sheath blight fungus, and the other for the sheath blot fungus. Sheath blight, caused by *Thanatephorus cucumeris*, is a notorious disease of rice with serious losses every year [18]. *Ceratobasidium oryzae-sativae *is another pathogen of rice, which causes sheath blot. These two pathogens both have their anamorphs (asexual stage) in the fungal form-genus *Rhizoctonia*, but are considered distantly related. Further testing with the fungal strain from the field using species-specific primers, Southern hybridization, DNA cloning, and DNA sequencing yielded results implying that gene transfer had occurred between rDNA genes of these two species of pathogenic fungi, and that genetic recombination had occurred at the 5.8S gene.

## Results and Discussion

**Figure 1:**
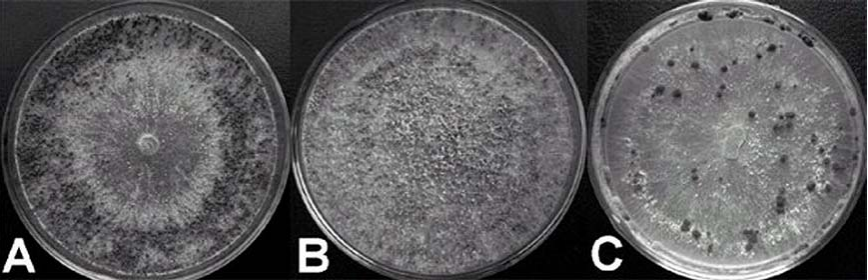
Colony morphology of (A) *Ceratobasidium oryzae-sativa *WH-87, (B) Strain RCOL-1 and (C) *Thanatephorus cucumeris *WH-1, showing abnormal distribution of sclerotia of strain RCOL-1 when grown at 25°C on PDA for 10 days.

**Figure 2:**
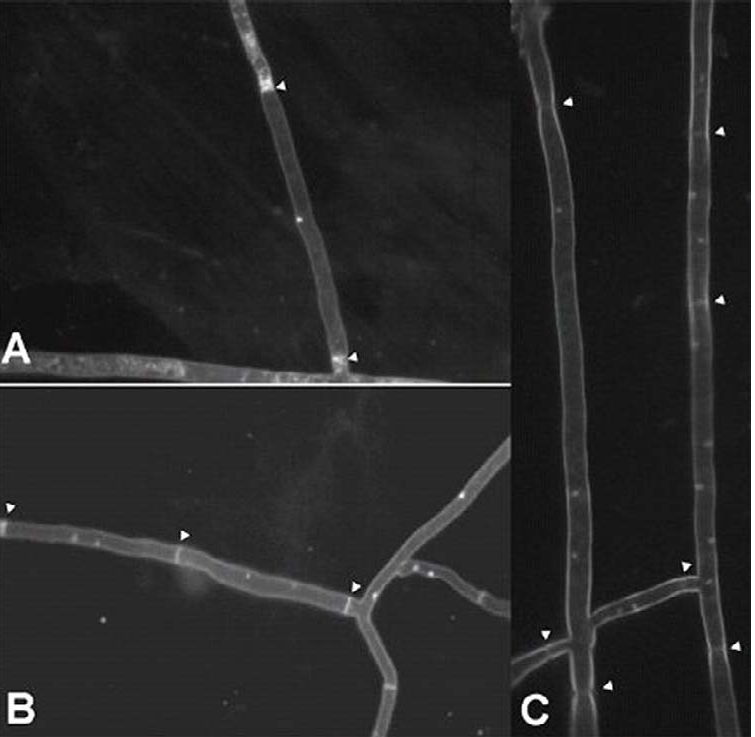
DAPI-stained nuclei in hyphae of strain RCOL-1: (A) only one nucleus in one cell; (B) two nuclei per cell; and (C) two or more nuclei per cell.

In November 2004, a fungal strain, named RCOL-1, was isolated from a typical rice sheath blight lesion in a field at Huazhong Agricultural University in Central China. The growth rate of RCOL-1 was slower than the average for either of the two major fungal rice pathogens *Thanatephorus cucumeris *or *Ceratobasidium oryzae-sativae*. This isolate also showed abnormal colony formation on PDA (potato dextrose agar), with sclerotia irregularly distributed compared to the two rice pathogens (Fig 1). RCOL-1 was also found to be avirulent when inoculated onto plant host tissue. DAPI staining (4',6-diamidino-2-phenylindole stain, [19]) showed that the distribution of nuclei in individual cells of RCOL-1 was not uniform (Fig 2), with some cells containing only one nucleus (1.3% out of 1210 cells counted), some with two nuclei (95.9%), and some multinucleate (2.8%) with up to 5 nuclei observed per cell. In contrast, cells of *T. cucumeris *consistently contain multiple nuclei, and *C. oryzae-sativae *has two nuclei in each cell [20].

**Figure 3:**
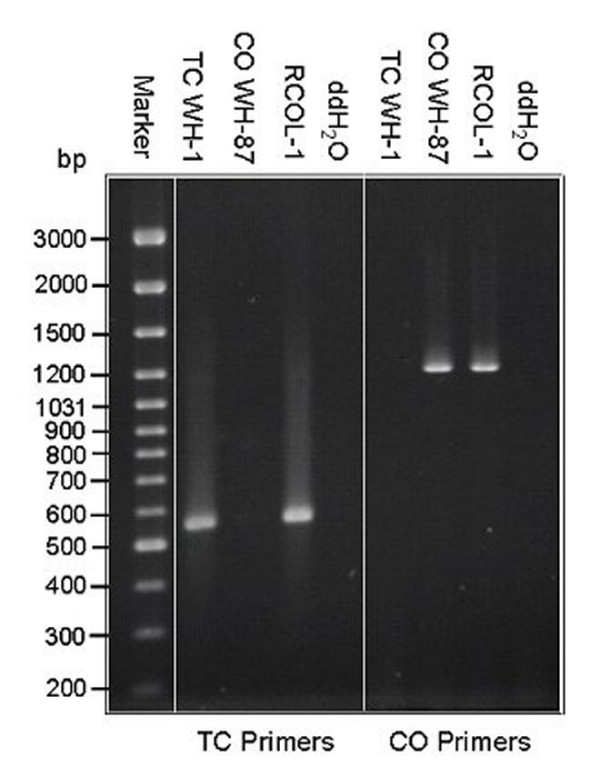
**PCR identification of strain RCOL-1 with specific primer pairs for *Thanatephorus cucumeris *(TC Primers: P-ITS1 and GMRS-3) and for *Ceratobasidium oryzae-sativa *(CO primers: GMROS and R635).** The genomic DNA of strain WH-1 of *T. cucumeris *(TC WH-1) and strain WH-87 of *C. oryzae-sativa *(CO WH-87) were used as controls; ddH_2_0 was used instead of DNA as reaction control. DNA weight marker is shown on the left.

**Figure 4:**
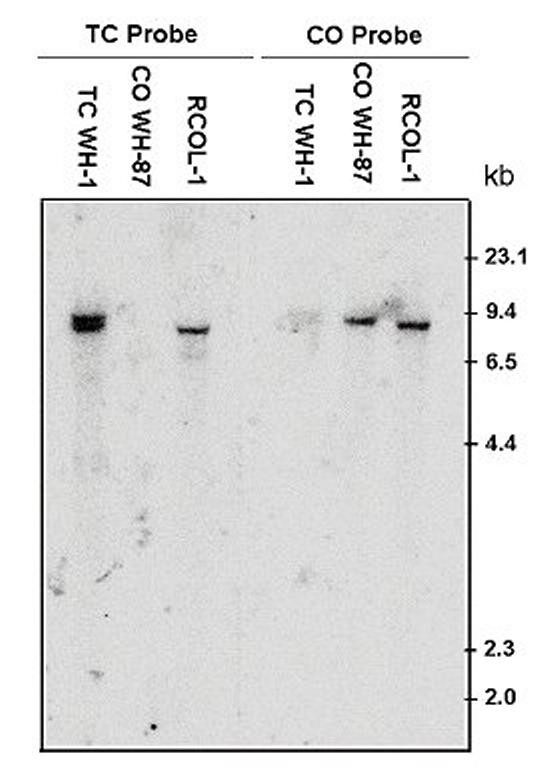
**Southern blot analysis of the ITS DNA of strain RCOL-1.** Oligonucleotides derived from the ITS1 sequences of *Ceratobasidium oryzae-sativa *(CO-Probe) or that of *Thanatephorus cucumeris *(TC-Probe) were 5'-^32^P labeled, and used as probes. Strain WH-1 of *T. cucumeris *(TC WH-1) and strain WH-87 of *C. oryzae-sativa *were used as controls. The genomic DNA samples were digested completely with *Bam*H.

DNA of RCOL-1 was tested using primers specific for either *T. cucumeris *or *C. oryzae-sativae *[18] based on the internal transcribed spacer (ITS) of the multi-copy nuclear ribosomal DNA genes (rDNA). RCOL-1 was found to have target bands of both fungal species (Fig 3). Furthermore, Southern blot of genomic DNA showed that the DNA of RCOL-1 could hybridize with either of the two probes, but the DNA of isolate WH-1 (*T. cucumeris*) or WH-87 (*C. oryzae-sativae*) would only hybridize with their respective probes (Fig. 4). These results confirmed that RCOL-1 contained rDNA sequence from both *C. oryzae-sativae *and *T. cucumeris*.

**Figure 5:**
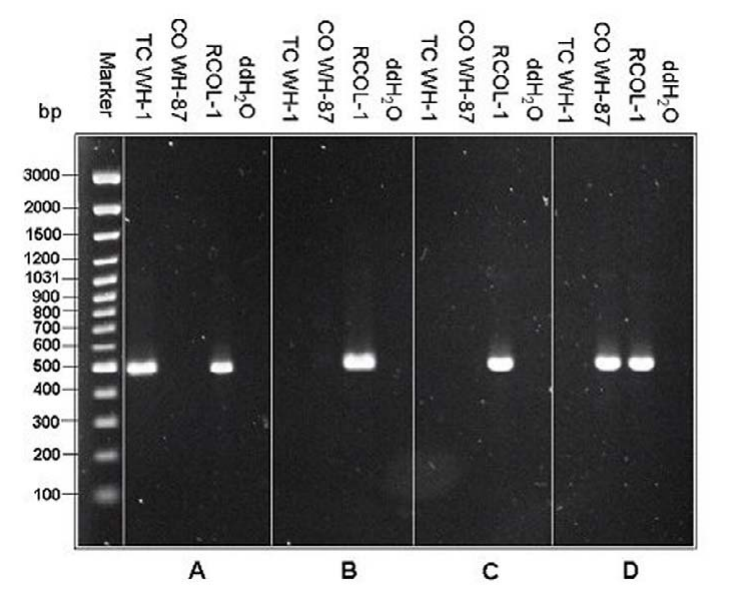
**Strain RCOL-1 contains four types of ITS DNA: (A) DNA amplified with P-RSITS1-F and GMRS-3; (B) DNA amplified with P-RSITS1-F and P-COITS2-R; (C) DNA amplified with P-COITS1-F and GMRS-3; and (D) DNA amplified with P-COITS1-F and P-COITS2-R.** DNA weight marker is shown on the left.

In addition to primer GMRS-3 which was based on the ITS2 sequence of *T. cucumeris *[18], three other primers were used which were designed from the ITS1 sequence of *T. cucumeris *(P-RSITS1-F), or the ITS1 (P-COITS1-F) or ITS2 (P-COITS2-R) of *C. oryzae-sativae*. The two forward and two reverse primers were used in all combinations. The amplification products (Fig 5) were sequenced, and these showed that RCOL-1 contained four types of ITS sequences, ones fully matching either *C. oryzae-sativae *(GenBank Accession DQ307249) or *T. cucumeris *(DQ307250), and two types of chimeric sequences: ITS1 DNA from *C. oryzae-sativae *with ITS2 DNA from *T. cucumeris *(DQ307251) or ITS1 DNA from *T. cucumeris *with ITS2 DNA from *C. oryzae-sativae *(DQ307252). The 747 bp alignment of the ITS of *C. oryzae-sativae *(712 bp) with *T. cucumeris *(718 bp) showed 79.5% identity, further demonstrating that these are distinct species, although they both have their anamorphs in the form-genus *Rhizoctonia*.

Clones were generated from polymerase chain reaction (PCR) products derived using universal fungal primers, ITS1 and ITS4 [17], and these clones were tested with the four pairs of primers mentioned above. Among the 500 clones, 499 were found to contain a complete ITS cassette of *C. oryzae-sativae*, but one of the clones was chimeric, with ITS1 of *C. oryzae-sativae *and ITS2 of *T. cucumeris*, based on primer amplification and DNA sequencing. To specifically detect the presence of *T. cucumeris *DNA, genomic DNA of RCOL-1 was amplified with the primer pair ITS1 (non-specific) and GMRS-3 (specific for the ITS2 region of *T. cucumeris*). The PCR product was used to generate 66 clones which were tested with the primer pairs COITS-F and GMRS-3 (for chimeric *C. oryzae-sativae *ITS1 and *T. cucumeris *ITS2), and P-RSITS1-F and GMRS-3 (for pure *T. cucumeris *ITS), giving 49 and 17 clones, respectively. Similarly, DNA of RCOL-1 was amplified with the primer pair RSITS1 (specific for ITS1 region of *T. cucumeris*) and ITS4 (non-specific). Clones were obtained, and 59 were tested with the primer pairs P-RSITS1-F and P-COITS2-R (for chimeric *T. cucumeris *ITS1 and *C. oryzae-sativae *ITS2), and P-RSITS1-F and GMRS-3 (for pure *T. cucumeris *ITS), giving 46 and 13 clones, respectively. The ratio of chimeric clones to pure *T. cucumeris *clones in each test was between 3 to 3.5 implying that there are more copies of the chimeras than complete *T. cucumeris *ITS sequences. The greater abundance of chimeric sequences than pure *T. cucumeris *ITS sequences may be a result of crossing over events at the conserved 5.8S gene, which sits between ITS1 and ITS2.

Hyphae of *T. cucumeris *and *C. oryzae-sativae *are vegetatively incompatible, and do not successfully anastomose. However, they parasitize the same host, and perhaps under the influence of host enzymes targeted to weaken pathogen cells or in dying host plant tissue, the fungal hyphae lost their integrity, and normal vegetative incompatibility mechanisms were overcome, allowing the hyphae to fuse. This direct intimate contact within the host could theoretically have lead to a transfer of genomic DNA between the fungi. Unlike plants and animals, vegetative cells of fungi are less differentiated, and each cell has the ability to develop into a new mycelium. This means that if any fungal cell were to obtain foreign DNA that was compatible with the DNA replication system, the cell could theoretically propagate the foreign DNA to daughter mycelia.

HGT was thought to be limited to the early stages of diverging lineages [21], although a review of more recent literature [2] has found frequent HGT in some phagotrophic algae and HGT between plant lineages, with few examples for animals or fungi. The HGT event between *C. oryzae-sativa *and *T. cucumeris *which generated RCOL-1 was probably a relatively recent one, since such an abnormal isolate may be less fit and not have a high survival rate in nature. In July 2005, eight months after the initial isolation of RCOL-1, more intensive sampling was conducted in the field where RCOL-1 was originally collected, and three other strains with morphology similar to RCOL-1 were found, although not at the original site of collection. This suggests that isolates such as RCOL-1 might be able to persist, even though macroscopically avirulent in pathogenicity tests, and with less aggressive growth than either parental type. Another explanation is that the intergeneric hybridization events occurred more than once in this field, and are more frequent than currently thought.

## Conclusion

We hypothesize that a presumably rare anastomosis event between hyphae of *T. cucumeris *and *C. oryzae-sativae *allowed the transfer of nuclei into a *C. oryzae-sativae *mycelium, followed by nuclear fusion and crossing over events. The relative scarcity of *T. cucumeris *ITS sequences compared to *C. oryzae-sativae*, could be a result of the transfer of just a few nuclei from *T. cucumeris *into an extensive *C. oryzae-sativae *mycelium, or the subsequent process of concerted evolution (Elder and Turner 1995) to homogenize the ribosomal gene sequences. Species of *Rhizoctonia *are known to be multinucleate, but the presence of chimeric forms of the ITS in RCOL-1 indicates that DNA of the two parental species had been mixed, rather than existing solely in separate nuclei. Furthermore, the growth debilitation and avirulence found with RCOL-1 are not likely to have resulted just from the altered ribosomal gene cassettes, since there are mostly pure copies of *C. oryzae-sativae *in RCOL-1, which should be sufficient for full function. There are probably other genes in RCOL-1 that have been affected by the intergeneric hybridization, giving the debilitated phenotype. Further research remains to reveal which other genes may have been affected, but the results of the current study indicate that horizontal gene transfer can occur between different genera of fungi, and based on the presence of other similarly debilitated field isolates, we speculate that such occurrences may not be so rare.

## Methods

### Strains and medium

RCOL-1, WH-1 and WH-87 were isolated from typical sheath blight lesions in a rice field at Huazhong Agricultural University, Wuhan, China. These isolates were subcultured numerous times from hyphal tips to ensure purity of each isolate. RCOL-1 grew slowly on PDA with abnormal colony morphology, while WH-1 and WH-67 were identified as normal strains of *Thanatephorus cucumeris *and *Ceratobasidium oryzae-sativae *respectively.

### DAPI staining of nuclei

Strains were grown in PDA plates, and autoclaved glass slides were placed onto the agar. After hyphae grew onto the slides, a droplet of 0.004 % DAPI was placed on each slide, followed by incubation in the dark for 20 min. The nuclear type of each strain was observed with a Nikon fluorescence microscope fitted with an ultraviolet light filter.

### Primers and PCR amplification

Fungal mycelia were collected and subjected to DNA extraction using the CTAB method [22]. Amplification of Internal transcribed spacers (ITS) was performed using primers P-ITS1 (5'-TCCGTAGGTGAACCTGCGG-3') and P-ITS4 (5'-TCCTCCGCTTATTGATATGC-3') according to [17]. Specific primers for distinguishing *T. cucumeris *and *C. oryzae-sativae *were designed and used based on Johanson et al [18]: GMRS-3 (5'-AGTGGAACCAAGCATAACACT-3') and P-ITS1 were used to specifically amplify DNA from *T. cucumeris*; and GMROS (5'-GAAAGAGAGAGAGGTCGCCTC-3') and R635 (5'-GGTCCGTGTTTCAAGACGG-3') were used to specific amply the DNA from *C. oryzae-sativae*.

To further analyze the PCR products of ITS DNA of RCOL-1, in addition to primer GMRS-3 (as a reverse primer for the ITS2 region of *T. cucumeris*), three more primers were designed based on the ITS sequence of *C. oryzae-sativae *and *T. cucumeris*: P-COITS1-F (5'-CCTTTCCTCCCAGG-3'), P-COITS2-R (5'-GGAACCAAGTTCATAGA-3') and P-RSITS1-F (5'-TCTACCTTAATTTGGC-3'). The two forward primers were paired with the two reverse primers giving four possible pairings. The genomic DNA of RCOL-1 was amplified with P-ITS1 and P-ITS4, and the PCR product was purified and diluted 100 fold (about 1.0 ng/μl) for second round amplification with the four pairs of primers. Amplification (25 μl) were performed using 1.0 μl purified PCR product, 2.5 μl of 10 × PCR buffer, 2.5 μl of 2.5 mM dNTPS, 1.0 μl of 1.5 μM forward primer and 1.0 μl of 1.5 μM reverse primer, 0.25 μl of 5.0 U/μl Taq (TAKARA, Dalian, P R China). A PCR amplification system (PTC-100: MJ, USA) was used with the following programme: initial denaturation for 4 min at 94°C, 30 s at 94°C, 30 s at 56°C, 30 s at 72°C, 20 cycles; 7 min at 72°C. PCR products were separated by electrophoresis on 1.2 % (w/v) agarose gel, and stained in ethidium bromide.

### Southern hybridization

To confirm the result obtained by PCR amplification, genomic DNA of strain RCOL-1, *T. cucumeris *strain WH-1 and *C. oryzae-sativae *WH-87 was extracted in CTAB following [22], and 15 μg of DNA of each strain was digested completely with *Bam*H1, which does not have any recognition sites on the ITS or 5.8 S rDNA of these two fungi. The digested products were size-fractionated through a 0.8 % agarose gel and mounted onto positively charged nylon membranes. Two oligonucleotides, namely TC-Probe (5'-CAATAGTTGGTGGATTTAATTCCATCATCCATTTGCTGT-3') and CO-Probe (5'-TTTAACTAGGGAAAGAGAGAGAGGTCGCCTCCGTCTA-3') which were derived from the ITS1 sequence of *T. cucumeris *or *C. oryzae-sativae*, respectively, were synthesised and 5'-labelled with γ-32P ATP by T4 polynucleotide kinase as probes to hybridize with DNA of RCOL-1. Hybridization was conducted in a hybridization incubator (Biorad, USA). Briefly, the membranes were pre-hybridized in 3×SSC containing 0.1 % SDS (sodium dodecyl sulfate) at 65°C for 7 h in a hybridization tube, and then probes were added into the hybridization buffer. Hybridization was carried out at 65°C overnight. To remove non-hybridized probes, membranes were washed in 100 ml of 6×SSC containing 0.1 % SDS three times for 15 min, then in 100 ml of 2×SSC containing 0.1 % SDS for 10 min, and finally in 100 ml of 1×SSC containing 0.1 % SDS for 5 min. The temperature for washing was controlled at 60°C. The membranes were exposed to an imaging plate and the locations of the labelled compounds were visualized by using a Molecular Dynamics (Sunnyvale, CA, USA) Strom-820 imaging-system analyzer.

### DNA sequencing

PCR products at various stages of this study were fractionated by electrophoresis in agarose gels and purified using a Gel Extraction Kit (Sangon, Shanghai, P R China). The recovered DNA was ligated onto T-Vector (pMD-18, TAKARA, Dalian, P R China), and then transformed into competent cells of *E. coli *JM109. Sequencing was done by the dideoxy-nucleotide termination method using the Big Dye terminator Sequencing Kit (BigDye terminator v2.0) and an ABI PRISM 377-96 automated sequencer (Sangon, Shanghai, P.R. China). The M13 universal primer was used for sequencing.

## List of Abbreviations

bp – Base pair, CTAB – Cetyl trimethylammonium bromide, DNA – Deoxyribonucleic acid, HGT – Horizontal gene transfer, ITS – Internal transcribed spacer region of rDNA, PCR – Polymerase chain reaction, rDNA – Ribosomal DNA, rRNA – Ribosomal RNA, SDS – Sodium dodecyl sulphate, SSC – A solution consisting of 0.15 M sodium chloride and 0.015 M trisodium citrate

## Authors' contributions

DJ, YF, TH and YP designed the experimental strategy; JX, YF, BL, DJ conducted the experiments; DJ, YF, JX, TH, YP, GL and JH were involved in the data analysis and their processing; and DJ and TH wrote the manuscript. All authors approve of the final manuscript.
